# Association between Periodontal Treatment and Healthcare Costs in Patients with Coronary Heart Disease: A Cohort Study Based on German Claims Data

**DOI:** 10.3390/dj10070133

**Published:** 2022-07-13

**Authors:** Katja Blaschke, Martin Hellmich, Christina Samel, Stefan Listl, Ingrid Schubert

**Affiliations:** 1 PMV Research Group, Faculty of Medicine and University Hospital Cologne, University of Cologne, 50931 Cologne, Germany; ingrid.schubert@uk-koeln.de; 2 Institute of Medical Statistics and Computational Biology (IMSB), Faculty of Medicine and University Hospital Cologne, University of Cologne, 50931 Cologne, Germany; martin.hellmich@uni-koeln.de (M.H.); christina.samel@dguv.de (C.S.); 3 Department of Dentistry—Quality and Safety of Oral Healthcare, Radboud University—Radboudumc (RIHS), 6525 EX Nijmegen, The Netherlands; stefan.listl@radboudumc.nl

**Keywords:** periodontal disease, periodontal treatment, coronary heart disease, healthcare costs, claims data, periodontology, cardiology

## Abstract

There is empirical evidence of an association between periodontitis and coronary heart disease (CHD). However, it is uncertain whether periodontal treatment in CHD patients might lead to reduced healthcare costs. This study aims to assess the association between periodontal treatment and healthcare costs in newly diagnosed CHD patients. Data from 21,263 adults who were continuously insured between 2011 and 2016 and who were newly diagnosed with CHD in 2013 were selected from a German claims database. The study population was differentiated by the utilization of periodontal treatment. The average treatment effect (ATE) of periodontal treatment on healthcare costs (total, inpatient, outpatient, drugs) was investigated using weighted Poisson regression models conditional on covariates and is shown as a ratio (of geometric means). Periodontal treatment was documented for 4.7% of the persons in the study population. Newly diagnosed CHD patients showed an ATE of 0.98 for total healthcare cost (95% CI 0.90–1.06), 0.79 for inpatient costs (95% CI 0.61–1.04), and 0.95 for drug costs (95% CI 0.87–1.04). A statistically significant 7% increase in outpatient costs was shown (95% CI 1.01–1.13). Despite a lack of statistical significance in most cases, the study provides evidence of a meaningful decrease in inpatient costs after periodontal treatment. Further studies are needed.

## 1. Introduction

Periodontitis and coronary heart disease (CHD) are highly prevalent non-communicable diseases [1,2]. In 2019, 13.1% of the world population suffered from a severe form of periodontitis [3]. In Germany, half of all 35- to 44-year-olds already suffer from moderate to severe periodontitis, while the proportion increases to 90% among older seniors (≥75 years) [4]. Globally, CHD prevalence can be estimated at 6.3% and increases by age [5]. In Germany, about 7% of women and 10% of men will develop CHD during their lifetime [6].

CHD and periodontitis are two multifactorial diseases with common risk factors, such as diabetes or smoking [7,8]. Furthermore, there is ample empirical evidence pointing to a direct association between periodontitis and CHD [9,10,11,12]. In particular, the relationship between periodontitis and atherosclerosis, one of the main causes of CHD, plays a major role [11,13]. The following mechanisms are discussed. First, periodontal bacteria and bacterial antigens can enter the circulation and can deposit on the endothelial cells as atherosclerotic plaques [10,14]. Second, a high molecular similarity between endogenous proteins and periodontal antigens is discussed, leading to an inflammatory response and subsequently to endothelial injury [15,16]. Third, both periodontitis and CHD have a similar inflammatory pathway leading to higher levels of C-reactive protein (CRP) and other inflammatory markers [10,14,17]. Following up on this, studies have shown that periodontal treatment reduces CRP levels as well as other inflammatory markers and leads to an improved endothelial function [10,11,18,19]. In this context, it can be assumed that periodontal treatment can reduce the risk of cardiovascular disease in general and adverse cardiac events in CHD patients [10,11].

Against the background of high prevalence and an aging population, both periodontitis and CHD cause a high financial burden for the healthcare system [20,21]. Whether the positive effect of periodontal treatment on the course of disease in CHD patients can lead to a reduction in healthcare costs is still unclear. The current literature is very sparse and contradictory [22,23]. While Albert et al. [22] showed an increase in healthcare costs in CHD patients with periodontal treatment, Jeffcoat et al. [23] reported a reduction in healthcare costs and a decrease in hospitalizations. Further studies are needed to address this knowledge gap.

To this end, the present study investigated the association between periodontal treatment and different kinds of healthcare costs in patients newly diagnosed with CHD. We hypothesized that periodontal treatment will significantly reduce healthcare costs in patients with CHD.

## 2. Materials and Methods

We conducted a retrospective cohort study. The full dataset of the InGef (Institute for Applied Health Research Berlin) research database was used. The InGef research database includes longitudinal health insurance data on at least six million Germans insured with about 70 statutory health insurers, mainly company health insurance providers [24]. Data for the years 2011 to 2016 were provided and the following profiles were available: master data (e.g., age, sex, insurance period, federal state), outpatient data with quarterly coded diagnoses (ICD-10, 10th revision, German modification (GM)) and codes for physician groups, inpatient data (date of admission/discharge, ICD-10-GM codes), as well as data on remedies and medical aids, drugs, dental services, and healthcare costs. Ethical approval was not required for this study. The data were available pseudonymized and were analyzed retrospectively, so that a subsequent assignment to sensitive patient data was not possible. 

The plausibility check showed negative or missing costs in the inpatient sector. Costs were imputed for the corresponding inpatient cases according to the following procedure: Based on inpatient cases from the entire InGef research database for which cost data were available, mean costs were calculated per year and per Diagnosis Related Group or, if no Diagnosis Related Group code was available, per treatment type for one hospital day. For inpatient cases with missing/negative costs the calculated mean costs were extrapolated to the respective length of stay.

### 2.1. Study Population

**Figure 1:**
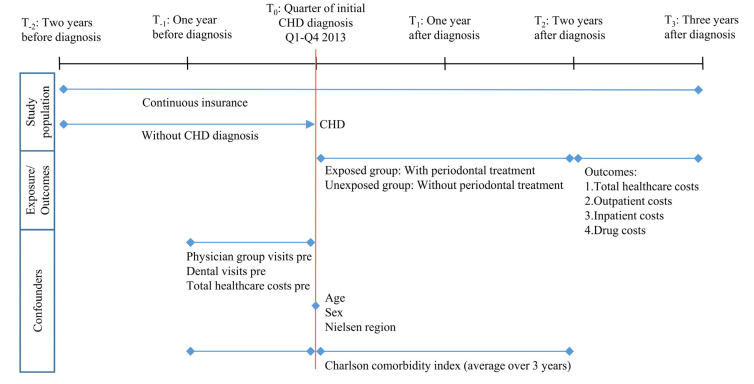
Study timeline and variables definition. Q1: first quarter of the year. Q4: fourth quarter of the year. Pre: one year before index quarter.

The study population comprises individuals who were continuously insured between 2011 and 2016, were at least 18 years old in 2013, and were newly diagnosed with CHD in 2013 (Figure 1). CHD was detected using ICD-10-GM codes I20 to I25, Z95.1, and Z95.5. The first diagnostic quarter in 2013 was defined as the index quarter. CHD diagnoses were internally validated [25]. An insured person was defined as having CHD if (i) there was an inpatient discharge diagnosis in 2013, or (ii) there was an inpatient secondary diagnosis or an outpatient diagnosis (modification “G” [assured]) in the index quarter and another outpatient diagnosis (modification “G”, “Z” [condition after]) within three quarters after the index quarter, or (iii) an outpatient diagnosis (modification “G”) was documented in the index quarter and an inpatient secondary diagnosis was present within three quarters after the index quarter. An incident case was given if no outpatient (modification “G”, “Z”) or inpatient secondary or discharge diagnosis for CHD was documented for the additional eight quarters prior to the index quarter.

### 2.2. Exposure to Periodontal Treatment

The study population was subdivided into an exposed and unexposed group. The exposed group included all individuals from the study population who had received at least one periodontal treatment in the index quarter or the eight subsequent quarters. The unexposed group was defined as having had no periodontal treatment in the index quarter or the following two years. The treatment fee codes P200-P203, 108, and 111 according to the German uniform assessment standard for dental services (BEMA) were used to determine the periodontal treatments (Table S1) [26].

### 2.3. Outcome

In this study, total healthcare costs from the viewpoint of statutory health insurance are used as the outcome and defined as the sum of inpatient, outpatient, and drug costs. Costs are expressed in Euro. The costs of each sector individually were also considered. In the outpatient sector, only costs from the area of human medicine, i.e., excluding dental costs, were taken into account.

### 2.4. Confounders

Potential confounders of the association between the utilization of periodontal treatment and healthcare costs were identified as health status, motivation to seek healthcare, and area of residence, in addition to age (in the index quarter) and sex. Based on the available data, the variables were operationalized as follows: Health status via Charlson comorbidity index, and motivation to seek healthcare via (i) total healthcare costs one year before the index quarter (sum of inpatient, outpatient, drug costs, costs of remedies/medical aids), (ii) number of physician groups visited one year before the index quarter (excluding dentists), and (iii) dental visits (yes/no) one year before the index quarter (Figure 1).

The information on area of residence was available at the federal state level. Due to low case numbers in some federal states, a regional allocation to Nielsen regions was made [27]. The Charlson comorbidity index [28] was defined as the average value over three years (one year before and two years after the index quarter). The algorithm of Quan et al. [29] was adopted, and internally validated inpatient and outpatient diagnoses (modification “G”, “Z”) were used. The diagnosis of myocardial infarction (ICD-10-GM: I21, I22, I25.2) as part of the definition of the target disease CHD was not included in the calculation of the Charlson comorbidity index.

### 2.5. Statistics

To examine the association between periodontal treatment and healthcare costs, the average treatment effect (ATE) was computed. A counterfactual framework was applied. Two potential outcomes are assumed for each individual; one with and one without periodontal treatment. However, in this cohort study, an individual can only be assigned to the exposed or unexposed group. That is why the counterfactual outcome of individual A was defined by searching the dataset for an individual B with comparable characteristics and different treatment to individual A. The exchangeability of the individuals is assumed [30]. The difference of the potential outcome means of the group with and without periodontal treatment results in the ATE. The statistical approach used in our study is based on the work of Nasseh et al. [31] and has already been applied by us in a population with incident diabetes [32].

Descriptive and multivariate analyses were performed. As the main multivariate model, a doubly robust (DR) method was chosen. The DR method builds on the combination of outcome regression and inverse probability weighting methods. Based on a weighted Poisson regression model conditional on the covariates for both the exposed and unexposed group, the predicted values for the whole sample were obtained [30]. The mean of the predicted values results in the potential outcome mean of the exposed and unexposed, respectively, from which the ATE was calculated. The related 95% confidence intervals (CI) were obtained by bootstrapping (1000 samples). The balance of the covariates between the exposed and unexposed groups after sample weighting was investigated by standardized differences. Based on the literature, a value of 0.1 was defined as the threshold, since at this value a relatively good balance of the covariates can be presumed [31,33].

For multivariate analysis, all costs were log transformed as a result of a right-skewed distribution (log (cost + 1)). For better understanding, the ATE results were back-transformed by an exponential function and displayed as a ratio (of geometric means). For comparison, a simple linear regression, an inverse probability weighted Poisson regression, as well as a Poisson regression conditional on the covariates were also conducted. The significance level alpha was set to 0.05 for all analyses. All analyses were performed using SQL and SAS 9.4 (SAS Institute, Cary, NC, USA).

## 3. Results

In this study, 21,263 insured persons newly diagnosed with CHD in 2013 could be included. Among these insured persons, the mean age was 64 years, 60.7% were men, and the average Charlson comorbidity index was 2 (Table 1). For slightly more than one-third of study participants, the place of residence was in North Rhine-Westphalia. One year prior to the index quarter, 70.4% of the insured persons received at least one dental treatment, an average of five different outpatient physician groups were visited, and the averaged total healthcare costs were 2518 €. Nearly 5% (*n* = 1003) of study participants could be assigned to the exposed group. Differences in exposed and unexposed group are most apparent with respect to dental care utilization before the index quarter and total healthcare costs before the index quarter. The exposed group visited dentists more often and had lower healthcare costs before the index quarter (Table 1). In addition, the exposed group was slightly younger on average and had a slightly lower Charlson comorbidity index. After reweighting the data, the values for the standardized differences (<0.1) indicated a good balance of covariates in the exposed and unexposed groups (Table S2).

With regard to the healthcare costs in the third year after the index quarter, the unexposed group showed higher amounts for almost all cost types (Table 2). In contrast, there were hardly any differences between the exposed and unexposed groups in terms of mean outpatient costs. When the geometric means were considered, the exposed group had lower costs in all sectors. Furthermore, it should be noted that for almost half of both the exposed and unexposed groups, no inpatient costs were documented in the third year after periodontal treatment. 

**Figure 2:**
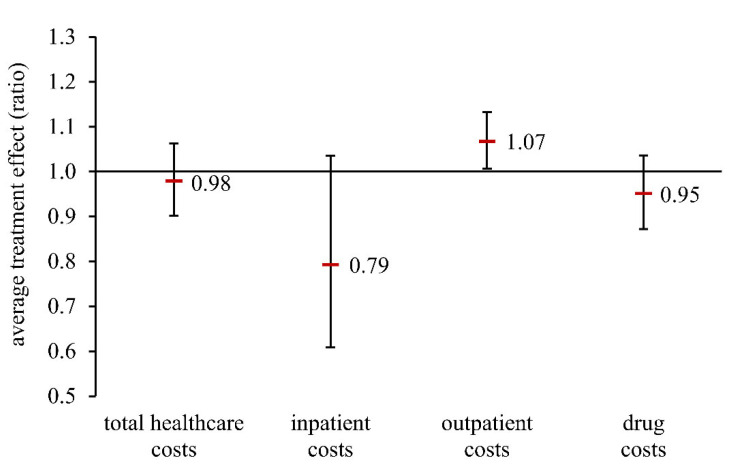
Average treatment effect (ratio of geometric means) in a population of patients newly diagnosed with CHD (doubly robust model). Total healthcare costs: sum of inpatient costs, outpatient costs, and drug costs.

An overview of the results on the average treatment effect (DR model) is presented in Figure 2. Insured persons in the exposed group had, on average, 0.98 fold the total healthcare costs (95% CI 0.90–1.06), 0.79 fold the inpatient costs (95% CI 0.61–1.04), and 0.95 fold the drug costs (95% CI 0.87–1.04) compared with the unexposed group. These results were not statistically significant. In contrast, an opposite and statistically significant effect could be seen for outpatient costs. Newly diagnosed CHD patients with periodontal treatment after initial diagnosis had outpatient costs which were 7% higher, on average, than the control group (ATE = 1.07, 95% CI 1.01–1.13). Comparison of the various model calculations yielded robust results (Table S3).

## 4. Discussion

This study analyzed the association between periodontal treatment and different kinds of healthcare costs in newly diagnosed CHD patients. Statistically significant results were only seen in outpatient costs. A slight increase in outpatient costs after periodontal treatment was evident. In contrast, a tendency towards more decreasing costs in the inpatient sector was observed, but without statistical significance. 

Our study is the first to investigate this topic based on German data in a population of CHD patients. However, the findings of our study cannot confirm the increase or reduction in total healthcare costs after periodontal treatment observed in two previous claims data studies. The US study by Jeffcoat et al. demonstrated a 10.7% annual reduction in medical costs (inpatient and outpatient) [23]. In contrast, the US study by Albert et al. [22] indicates a significant increase in medical costs in the group with periodontal treatment. In our study, no clear total healthcare cost savings or increase after periodontal treatment were revealed. Based on the confidence limits, our data show that periodontal treatment, on average, can result in anywhere from a 10% total healthcare cost reduction to a 6% increase. Comparison is hampered by the different healthcare systems and methodological differences. While all three studies are based on claims data analyses, there are differences regarding the study population. In the study by Jeffcoat et al., the definition of CHD did not include acute and old myocardial infarction (ICD-9 410, 412) [23]. Albert et al. [22] included considerably more medical conditions, such as heart failure or hypertension. Furthermore, both Albert et al. [22] and Jeffcoat et al. [23] included prevalent cases and the diagnoses were not internally validated. In our study, total healthcare costs additionally included drug costs next to inpatient and outpatient costs. In addition, our study considered more potential confounders in statistical analysis. 

For the study by Albert et al. [22], it is limiting to add that the exposure and the outcome were collected in the same two-year period. Thus, medical costs prior to the use of periodontal treatment may have been included in the analysis, which may bias the effect of periodontal treatment on medical costs. 

Furthermore, Jeffcoat et al. included only individuals with at least one periodontal treatment as diagnosed by a clinician. That means that patients with completed periodontal treatment (≥4 dental visits, treatment group) were compared with patients with three or fewer periodontal treatment visits (control group) [23]. In contrast, our study differentiated between periodontally treated and non-treated patients whose treatment costs were covered by statutory health insurance. The number of treatments performed was not considered in the grouping.

Besides reducing medical costs, Jeffcoat et al. demonstrated a 28.6% decrease in hospital admission rates in patients with CHD and periodontal treatment [23]. A cohort study from Taiwan also demonstrated reduced cardiovascular hospitalization rates after periodontal treatment in a population in end-stage renal disease and thus at increased risk for cardiovascular complications [34]. Although no statistically significant results were obtained, our data indicate an average reduction of 21% in inpatient costs after periodontal treatment. This reflects a relevant effect which should be investigated in further studies. The reduction of inpatient costs could also be due to fewer hospitalizations and a reduced need for intensive medical care. In this case, it would seem pertinent to consider oral health as a routine element in the prevention and management of CHD.

To our knowledge, the impact of periodontal treatment on outpatient or drug costs explicitly in CHD patients has not been investigated by other studies. While our data show a slight tendency towards decreasing drug costs in the absence of statistical significance, a reverse and statistically significant effect was seen for outpatient costs. Lower drug costs after periodontal treatment could be explained by the less frequent occurrence of adverse cardiac events and the positive effect of periodontal treatment on the whole complex of metabolic disorders [35], which could also reduce the need for medication. One potential reason for the observed higher outpatient costs could be that periodontal care also led to the closer inspection of general health and a more intensive course of physician care upon initiation by the dentist. According to the current German guidelines for the treatment of periodontitis, interventions to reduce periodontal risk factors, such as diabetes and smoking, are also recommended as part of comprehensive periodontal treatment [36]. Nevertheless, the influence of periodontal treatment on drug and outpatient costs would have to be further investigated.

Our study confirms that compared to the high prevalence of periodontitis in Germany [4], especially among CHD patients [37,38], the utilization rates of periodontal treatment are very low [7]. In a German cohort study that examined CHD patients from 2009 to 2011, almost all patients suffered from periodontitis, of which 47% had a severe form [37,38]. Periodontitis in the early stages is not accompanied by pain; as such, dentists are rarely contacted due to a low level of suffering [39]. Accordingly, physicians from the non-dental setting, such as general practitioners, internists, and cardiologists, should be urged to point out the existing relationship between periodontitis and CHD, to provide their patients with comprehensive information, and to advise their patients to visit a dentist if necessary [10]. A simple, user-friendly, and non-invasive screening tool could help to detect periodontitis at an early stage in the non-dental setting [39,40]. In addition to the non-dental setting, dentists are also called upon to ask patients about pre-existing conditions such as CHD, to explain the link between periodontitis and CHD, and to point out the need for careful oral hygiene at home, especially in the case of existing chronic conditions [10]. In this context, a closer collaboration between the dental and medical professions is urgently needed. Clinical decision support tools can help health professionals to identify risk patients early and to improve patient care by integrating user-friendly screening tools and providing individualized, evidence-based treatment recommendations [41]. The recently developed clinical decision support tool by the Dent@Prevent consortium already focuses on diabetes and periodontitis [41]. An extension for CHD is recommended. 

If periodontitis is detected at an early stage, the burden of disease could already be reduced by regular professional tooth cleanings and adequate individual oral hygiene [42]. The dental costs in this context would be small compared to the potential total healthcare costs due to acute cardiac events, complications of other chronic diseases, and the consequences of severe periodontitis with tooth loss. Although our study shows only a tendency of decreasing inpatient and drug costs as well as a slight increase in outpatient costs, larger cost savings could be expected. However, professional tooth cleaning in Germany is a private service, whereby the statutory health insurance policies offer different partial subsidies. Full coverage of professional tooth cleaning for CHD patients in Germany would increase the utilization rate and could complement the secondary prevention.

Our study has strengths and limitations. Claims data have the advantage of providing a large and longitudinal dataset that allows for the cohort study design, and rare events can also be detected [11]. Furthermore, claims data are free from recall and non-responder bias and include data from all insured individuals regardless of the accessibility for studies (no drop out) [43].

In contrast, the following limitations must be considered. First, our study is based on data mainly from company health insurance policies and the study population was restricted to patients continually insured between 2011 and 2016. That means that individuals who changed health insurance policies during this period (mostly younger individuals) or died (mostly older individuals) could not be considered. 

Second, claims data are collected for billing purposes. In Germany, a considerable proportion of professional tooth cleaning is being provided outside of statutory health insurance coverage and such utilization is not included in our claims data. Professional tooth cleaning may already be sufficient for the treatment of mild periodontitis [42]. Evidence from Korea suggests that regular dental visits for professional cleaning (once a year or more frequently) reduces cardiovascular risk by 14% [44]. In addition, information on individual oral hygiene behavior (e.g., tooth brushing) was not available. To account for these unobservable confounders, we included proxies such as the number of physician groups visited, dental visits, and the total healthcare costs one year before the initial CHD diagnosis. In addition, other unexplored variables might influence the oral environment. For example, the use of probiotics [45] or herbal compounds [46] could potentially alter clinical and microbiological parameters in patients with periodontal disease. Further studies are encouraged to consider such aspects in terms of their potential effects on chronic diseases and overall healthcare costs.

Third, the oral health status of individuals is not available in German claims data and so neither the diagnosis of periodontitis nor information regarding the severity of the disease was available to us. However, the lack of diagnosis is not a major problem in this study, as the costs of periodontal treatment are only covered by the statutory health insurance if there is evidence of a periodontal disease. Given the high prevalence of periodontitis in Germany and the known association between periodontitis and CHD [4,9,10,11,12], a high number of individuals with periodontitis can also be expected for the unexposed group. However, it cannot be excluded that our unexposed group also includes periodontally healthy patients. This could cause a bias towards the null. Likewise, based on the available data, we do not know whether periodontitis has been successfully treated. Due to a new German directive for the systematic treatment of periodontitis [47] which came into force in 2021, more periodontal treatment codes can be billed via statutory health insurance. This includes supportive periodontal care, which indicates that active periodontal treatment has been completed. Accordingly, based on these claims data, future studies can make more accurate statements about the completion of periodontal treatment.

Fourth, our study assessed outcome costs in the third year after the initial diagnosis of CHD. In the two previous years, the utilization of periodontal treatment was classified. We hypothesize that the elimination of periodontitis has a protective cardiovascular impact in our observation period, via the mechanisms described above. Nevertheless, it is possible that a longer period between exposure and outcome would reveal relevant cost effects due to fewer adverse cardiac events. This should be considered in further studies. 

Finally, because of the “small” exposed group and the imbalance between the exposed and unexposed groups, we may have failed to demonstrate statistically significant treatment effects in most cases.

Due to the limitations, our findings can only be generalized with caution.

## 5. Conclusions

In conclusion, due to a low utilization of periodontal treatments by insured persons, the results of our study are not sufficiently robust. In most cases, no statistically significant effects could be demonstrated. Prospective studies with higher numbers of exposed cases are needed. However, our study provides evidence of meaningful decreasing inpatient costs after periodontal treatment.

## Figures and Tables

**Table 1:** Baseline characteristics of the study population, in total and differentiated by those exposed and unexposed to periodontal treatment.

			Periodontal Treatment	
		Total	Exposed Group	Unexposed Group
**N** (%)		21,263 (100)	1003 (4.7)	20,260 (95.3)
**Sex**, *n* (%)	Men	12,903 (60.7)	646 (64.4)	12,257 (60.5)
	Women	8360 (39.3)	357 (35.6)	8003 (39.5)
**Age**, mean (SD)		64 (12.5)	60 (10.5)	64 (12.5)
**Nielsen region**, *n* (%)	Bavaria	2926 (13.8)	132 (13.2)	2794 (13.8)
	Baden-Wuerttemberg	2532 (11.9)	129 (12.9)	2403 (11.9)
	Centre	3472 (16.3)	131 (13.1)	3341 (16.5)
	North (West)	2772 (13.0)	125 (12.5)	2647 (13.1)
	North Rhine-Westphalia	7735 (36.4)	416 (41.5)	7319 (36.1)
	East (North)	1252 (5.9)	53 (5.3)	1199 (5.9)
	East (South)	545 (2.6)	16 (1.6)	529 (2.6)
	Missing	29 (0.1)	<5 *	<29 (0.1)
**Dental visit pre**, *n* (%)	Yes	14,963 (70.4)	825 (82.3)	14,138 (69.8)
	No	6300 (29.6)	178 (17.7)	6122 (30.2)
**Charlson comorbidity index**, mean (SD)		2.0 (1.9)	1.6 (1.7)	2.0 (1.9)
**Physician group visits pre**, mean (SD)		5.0 (3.0)	5.0 (3.2)	5.0 (3.0)
**Total healthcare costs pre**, mean (SD)		2517.8 (4882.3)	1998.5 (3660.1)	2543.5 (4933.6)

SD: standard deviation. Pre: one year before index quarter. Total healthcare costs pre: sum of inpatient, outpatient, drug costs, and costs of remedies/medical aids. * Percentage is not displayed if number of cases is <5. Percentage values are rounded to the first decimal place.

**Table 2:** Descriptive statistics of the different kinds of healthcare costs in the third year after the index quarter.

Costs	Periodontal Treatment	Min.	Q25	Median	Q75	Max.	Mean	Geom. Mean
Total healthcare costs, €	Exposed group	0	592	1136	2878	85,976	2950	1293
	Unexposed group	0	663	1295	3413	233,167	3488	1483
Inpatient costs, €	Exposed group	0	0	0	619	84,778	1643	12
	Unexposed group	0	0	0	1613	231,593	2051	17
Outpatient costs, €	Exposed group	0	412	685	1107	14,811	920	627
	Unexposed group	0	437	709	1136	25,543	921	656
Drug costs, €	Exposed group	0	72	160	403	19,053	386	169
	Unexposed group	0	86	210	527	80,618	516	174

Min.: minimum. Q25: 25% percentile. Q75: 75% percentile. Max.: maximum. Geom. mean: geometric mean. Total healthcare costs: sum of inpatient costs, outpatient costs, and drug costs.

## Data Availability

Restrictions apply to the availability of these data. Data was obtained from InGef and spectrumK GmbH under license for the current study, and so are not publicly available. The data analyses were carried on the premises of spectrumK, so that the data can be made available via spectrumK upon reasonable request and with permission of InGef and spectrumK GmbH.

## Supplementary Materials

The following supporting information can be downloaded at: https://www.mdpi.com/article/10.3390/dj10070133/s1, Table S1: Billing codes for periodontal treatment according to the German uniform assessment standard for dental services (BEMA); Table S2: Distribution of covariates after log transformation of costs and reweighting the data incl. standardized differences, differentiated by those exposed and unexposed to periodontal treatment; Table S3: Average treatment effect (ratio of geometric means) in patients newly diagnosed with CHD, all models.
