# Supplementary material for: Association between Periodontal Treatment and Healthcare Costs in Patients with Coronary Heart Disease: A Cohort Study Based on German Claims Data
Source: Dent J (Basel). 2022 Jul 13;10(7):133. doi: 10.3390/dj10070133 (PMC9320253; doi:10.3390/dj10070133)
Supplement: Supplementary file 1 [file dentistry-10-00133-s001.zip › dentistry-1776601-supplementary.pdf]

dentistry journal

MDPI

# *Supplementary Materials*

## 1 Supplementary Tables

**Table S1.** Billing codes for periodontal treatment according to the German uniform assessment standard for dental services (BEMA) [26].

| BEMA | Description |
|---|---|
| P200 | Systematic treatment of periodontal diseases (supra- and subgingival debridement), closed procedure per treated single-rooted tooth. |
| P201 | Systematic treatment of periodontal diseases (supra- and subgingival debridement), closed procedure per treated multi-rooted tooth. |
| P202 | Systematic treatment of periodontal disease (surgical therapy), open procedure per treated single-rooted tooth. |
| P203 | Systematic treatment of periodontal diseases (surgical therapy), open procedure per treated multi-rooted tooth. |
| 108 | Grinding in of the natural dentition for masticatory plane compensation and relief, per session. |
| 111 | Follow-up treatment as part of the systemic treatment of periodontal diseases, per session. |

**Table S2.** Distribution of covariates after log transformation of costs and reweighting the data incl. standardized differences, differentiated by those exposed and unexposed to periodontal treatment.

| | | Periodontal treatment | | |
|---|---|---|---|---|
| | | **Exposed group** | **Unexposed group** | **Standardized differences** |
| **Sex**, n (%) | | | | -0.0375 |
| | Men | 62.5 | 60.7 | |
| | Women | 37.5 | 39.3 | |
| **Age**, mean (SD) | | 63 (48) | 65 (13) | -0.0436 |
| **Nielsen region**, n (%) | | | | 0.028 |
| | Bavaria | 13.7 | 13.8 | |
| | Baden-Württemberg | 12.5 | 11.9 | |
| | Centre | 15.6 | 16.3 | |
| | North (West) | 13.2 | 13.1 | |
| | North Rhine-Westphalia | 36.4 | 36.4 | |
| | East (North) | 6.1 | 5.9 | |
| | East (South) | 2.7 | 2.6 | |
| **Dental visit pre**, n (%) | | | | 0.0352 |
| | Yes | 72.0 | 70.4 | |
| | No | 28.0 | 29.6 | |
| **Charlson comorbidity index**, mean (SD) | | 2.0 (9.1) | 2.0 (2.0) | -0.0057 |
| **Physician group visits pre**, mean (SD) | | 5.0 (14.0) | 5.0 (3.0) | 0.0103 |
| **Total healthcare costs pre** (log), mean (SD) | | 6.8 (7.2) | 6.8 (1.8) | -0.0047 |

SD: standard deviation. Pre: one year before index quarter. Total healthcare costs pre: sum of inpatient, outpatient, drug costs, costs of remedies/medical aids. Percentage values are rounded to the first decimal place.

**Table S3.** Average treatment effect (ratio of geometric means) in patients newly diagnosed with CHD, all models.

| | **Simple regression** | **Inverse probability weighting** | **Regression adjustment (Poisson)** | **Doubly robust method** |
|---|---|---|---|---|
| | ATE (95% CI) | ATE (95% CI) | ATE (95% CI) | ATE (95% CI) |
| Total healthcare costs | 0.87 (0.80, 0.95) | 0.96 (0.93, 1.00) | 0.99 (0.91, 1.08) | 0.98 (0.90, 1.06) |
| Inpatient costs | 0.72 (0.57, 0.92) | 0.79 (0.71, 0.87) | 0.82 (0.62, 1.07) | 0.79 (0.61, 1.04) |
| Outpatient costs | 0.98 (0.93, 1.04) | 1.06 (1.03, 1.08) | 1.07 (1.01, 1.13) | 1.07 (1.01, 1.13) |
| Drug Costs | 0.80 (0.73, 0.87) | 0.93 (0.90, 0.97) | 0.96 (0.88, 1.05) | 0.95 (0.87, 1.04) |

ATE: average treatment effect. CI: confidence interval. Total healthcare costs: sum of inpatient costs, outpatient costs, and drug costs.

## 2 Supplementary Reference

26. Kassenzahnärztliche Bundesvereinigung (KZBV); Spitzenverband Bund der Krankenkassen (GKV-Spitzenverband). *Einheitlicher Bewertungsmaßstab für zahnärztliche Leistungen gemäß § 87 Abs. 2 und 2h SGB V (BEMA): Anlage A zum Bundesmantelvertrag - Zahnärzte (BMV-Z)*; Germany: Cologne, Berlin, 2019.
